# Gamma-irradiated human amniotic membrane decellularised with sodium dodecyl sulfate is a more efficient substrate for the *ex vivo* expansion of limbal stem cells

**DOI:** 10.1016/j.actbio.2017.07.041

**Published:** 2017-10-01

**Authors:** G.S. Figueiredo, S. Bojic, P. Rooney, S.-P. Wilshaw, C.J. Connon, R.M. Gouveia, C. Paterson, G. Lepert, H.S. Mudhar, F.C. Figueiredo, M. Lako

**Affiliations:** a Institute for Genetic Medicine, International Centre for Life, Newcastle University, Central Parkway, Newcastle upon Tyne NE1 3BZ, UK; b Department of Ophthalmology, Royal Victoria Infirmary, Queen Victoria Road, Newcastle upon Tyne NE1 4LP, UK; c Tissue Services, NHS Blood and Transplant, 14 Estuary Banks, Speke, Liverpool L24 8RB, UK; d School of Pharmacy and Medical Sciences, Faculty of Life Sciences, University of Bradford, Bradford BD7 1DP, UK; e The Blackett Laboratory, South Kensington Campus, Imperial College, London SW7 2AZ, UK; f National Specialist Ophthalmic Pathology Service, Department of Histopathology, Royal Hallamshire Hospital, Glossop Road, Sheffield S10 2JF, UK

**Keywords:** Human amniotic membrane, Tissue decellularisation, Limbal stem cell culture, Brillouin microscopy

## Abstract

The gold standard substrate for the *ex vivo* expansion of human limbal stem cells (LSCs) remains the human amniotic membrane (HAM) but this is not a defined substrate and is subject to biological variability and the potential to transmit disease. To better define HAM and mitigate the risk of disease transmission, we sought to determine if decellularisation and/or γ-irradiation have an adverse effect on culture growth and LSC phenotype. *Ex vivo* limbal explant cultures were set up on fresh HAM, HAM decellularised with 0.5 M NaOH, and 0.5% (w/v) sodium dodecyl sulfate (SDS) with or without γ-irradiation. Explant growth rate was measured and LSC phenotype was characterised by histology, immunostaining and qRT-PCR (ABCG2, ΔNp63, Ki67, CK12, and CK13). Ƴ-irradiation marginally stiffened HAM, as measured by Brillouin spectromicroscopy. HAM stiffness and γ-irradiation did not significantly affect the LSC phenotype, however LSCs expanded significantly faster on Ƴ-irradiated SDS decellularised HAM (p < 0.05) which was also corroborated by the highest expression of Ki67 and putative LSC marker, ABCG2. Colony forming efficiency assays showed a greater yield and proportion of holoclones in cells cultured on Ƴ-irradiated SDS decellularised HAM. Together our data indicate that SDS decellularised HAM may be a more efficacious substrate for the expansion of LSCs and the use of a γ-irradiated HAM allows the user to start the manufacturing process with a sterile substrate, potentially making it safer.

**Statement of Significance:**

Despite its disadvantages, including its biological variability and its ability to transfer disease, human amniotic membrane (HAM) remains the gold standard substrate for limbal stem cell (LSC) culture. To address these disadvantages, we used a decellularised HAM sterilised by gamma-irradiation for LSC culture. We cultured LSCs on fresh HAM, HAM decellularised with NaOH, HAM decellularised with sodium dodecyl sulfate (SDS) and HAM decellularised with SDS and sterilised with gamma-irradiation. We demonstrated that although HAM decellularised with SDS and sterilised with gamma-irradiation is significantly stiffer this does not affect LSC culture growth rate or the phenotype of cultured LSCs. We therefore recommend the use of SDS decellularised gamma-irradiated HAM in future LSC clinical trials.

## Graphical abstract

**Figure:**
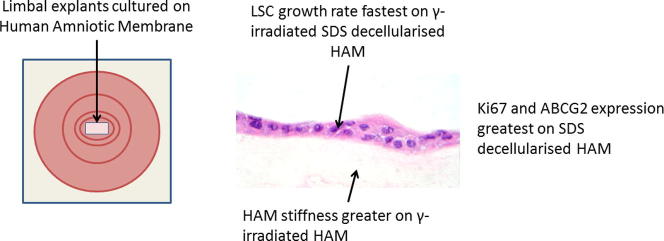

## 1 Introduction

Human amniotic membrane (HAM) has long been used in ophthalmic procedures to treat ocular surface diseases and burns, due to its ability to promote re-epithelialisation via growth factors (such as EGF, KGF and HGF) and its basement membrane [1], and to inhibit fibrosis through suppression of TGFβ signalling [2]. Fresh HAM consists of an epithelial layer (devitalised by the freezing process), a stroma and a thick basement membrane. The basement membrane and extracellular matrix components of HAM, when used as a substrate, have shown similar properties to conjunctival and corneal epithelium [3], [4].

Human limbal epithelial stem cells (LSCs) have commonly been expanded *ex vivo* on human amniotic membrane for both clinical and research purposes [5], [6], [7]. HAM shows low or no immunogenicity [8], [9], [10], [11]. It is not a defined substrate and has a number of disadvantages, most importantly its biological variability and the potential to carry or transmit infections, but despite this, HAM remains the gold standard and most widely used substrate for the expansion of LSCs *in vitro* and in clinical trials [12], [13], [14]. Recently, there has been a significant drive to better characterise HAM in order to develop a better substrate for the *in vitro* expansion of LSCs [13], [15], [16]. One such approach has been to decellularise HAM, as this process can remove all cellular, immunogenic components from a tissue, whilst preserving key extracellular matrix (ECM) components and the basement membrane, ensuring cell attachment and expansion. The decellularisation process may create a more consistent, defined substrate that does not elicit an adverse immunological response *in vivo* [17], [18], [19]. Several studies have suggested that LSCs cultured on decellularised HAM expand more quickly, but are also more differentiated compared to fresh HAM [20], [21], [22]. The improved cell proliferation has been attributed to better cell adhesion to the substrate compared to fresh HAM, due to exposure of the basement membrane, unobstructed by a devitalised epithelial cell layer.

Decellularisation of human tissues has been extensively investigated and applied to a wide array of tissues for different clinical applications. A wide range of agents have been used to decellularise tissues, including sodium dodecyl sulfate (SDS) [17], dispase, thermolysin, trypsin, ethylenediamine tetra-acetic acid (EDTA), and ethanol, among others [12], [23]. In 2013, Saghizadeh et al. described a new, simpler and faster decellularisation method that involved using a cotton bud soaked in sodium hydroxide 0.5 M solution to rub the epithelial side of the HAM to debride its epithelial cells in a period of five to ten seconds [12]. It was demonstrated that the HAM retained the native ECM structure following decellularisation with sodium hydroxide.

A characteristic of HAM is its biological variability, both in thickness and elasticity [24], [25]. In 2012, Jones et al. [26] applied a theory first demonstrated in 2006 by two separate groups that showed that human mesenchymal stem cells are sensitive to substrate rigidity and matrix elasticity which when altered can give rise to specific stem cell lineages [27], [28]. Jones et al. demonstrated that LSCs cultured on uncompressed (i.e. with reduced stiffness) collagen gels were less differentiated than those cultured on compressed collagen gels [26]. The same group showed that central cornea is stiffer than peripheral cornea, resulting in migration and differentiation of LSCs [29]. Molladavoodi et al. supported this finding when demonstrating that human corneal epithelial cells had lower rates of migration in compliant tissues [30]. It has therefore been hypothesised that the mechanical properties (stiffness) of the corneal anterior surface represents a major factor regulating corneal epithelium homeostasis [29]. In particular, it has been proposed that the mechanical environment of the limbus (i.e. being more compliant) may be fundamental for the maintenance of LSCs, whereas the stiffer matrix of the central cornea is instrumental in driving centripetal cell migration through durotaxis and inducing epithelial cell differentiation [29]. Lepert et al. used Brillouin microscopy as a reliable method of measuring corneal stiffness and demonstrated similar findings [31]. Chen et al. demonstrated that γ-irradiation can cross-link collagen-chitosan scaffolds [32]. Mi et al. showed that UVA cross-linked plastically-compressed collagen (PCC) gels had a greater breaking force than uncross-linked PCC [33]. We therefore wondered whether γ-irradiation and/or the decellularisation process could affect HAM stiffness.

Decellularisation may better define HAM, however, importantly for a tissue that will consequently be used in humans, the risk of disease transmission remains in a non-sterile substrate. Hogg et al. demonstrated in 2015 that γ-irradiation is an effective method of terminal sterilisation in the production of decellularised skin dermis for direct allogeneic transplantation [34]. Ƴ-irradiated HAM is readily available in the UK as a product provided by NHS Blood and Transplant (NHSBT), however, to the best of our knowledge, no previous work has been done regarding the potential effect of this sterilisation process on LSC growth *ex vivo*, and as HAM is the most widely used substrate in LSC culture, we aimed to determine whether γ-irradiation had any effect on LSC culture.

The aim of this study was to investigate the potential use of decellularised HAM (a better defined substrate) for the *ex vivo* expansion of LSCs. As such, we evaluated the proliferation and phenotype of LSCs grown on HAM decellularised by two different methods (i.e. 0.5 M NaOH and 0.5% SDS), and compared to frozen non-decellularised HAM. We assessed whether using γ-irradiation as a terminal sterilisation step in the production of a clinical-grade substrate would affect LSC proliferation and phenotype and thus produce a substrate at least as effective but safer as it reduces the risk of disease transmission. We also aimed to determine whether substrate stiffness is affected by γ-irradiation or the decellularisation process and whether this has an impact on LSC growth and differentiation.

## 2 Methods

All experimental protocols were previously approved by Newcastle University and research was conducted in accordance with the tenets of the Declaration of Helsinki.

### 2.1 Human amniotic membrane (HAM) sourcing

Cryopreserved HAM tissue (SDS decellularised and non-decellularised) was obtained frozen in 50% glycerol from NHS Blood and Transplant (NHSBT, UK) in 3 × 3 cm square sheets, wrapped around nitrocellulose paper with a service level agreement with Newcastle upon Tyne Hospitals NHS Foundation Trust, UK. All donors had consented for use of their tissues for research and the study was carried out in full accordance with our regional ethics committee approval and research agreement. SDS decellularisation was performed before freezing and NaOH decellularisation was performed after thawing frozen fresh (non-decellularised) HAM.

### 2.2 HAM decellularisation with 0.5% (w/v) SDS

Decellularisation was performed based on the procedure described by Wilshaw et al. [17]. Each samples of HAM was initially incubated in Cambridge antibiotic solution (Source Bioscience, UK) for 30 minutes at 37 °C. HAM samples were subjected to a single cycle of hypotonic buffer (10 mM Tris; pH 8.0) at 4 °C for 24 hours and hypotonic buffer containing 0.5% (w/v) SDS at 37 °C for 24 hours (pH 7.4) with agitation in the presence of protease inhibitors (Aprotinin, 10 KIU.mL^−1^ and 0.1%; (w/v) EDTA). Each sample was then incubated in incubated in DNAase (50 U.mL^−1^) and RNAase (1 U.mL^−1^;) in buffer (50 mM Tris-HCl, 10 mM MgCl2, 50 mg.mL^−1^ bovine serum albumin at pH 7.5) for three hours at 37 °C with gentle agitation. Finally, all samples were washed three times in PBS at 4 °C for 30 minutes each with agitation followed by two 24 hour washes. Half of the 0.5% SDS decellularised HAM were then γ-irradiated at a target dose of 25 kGy, an internationally-recognised sterilisation dose used by NHSBT to sterilise bone, tendons, skin and decellularised dermis for clinical use.

### 2.3 HAM decellularisation with 0.5 M NaOH

Fresh HAM obtained from NHSBT was thawed and washed in PBS, unwrapped from its nitrocellulose paper backing and stretched out on a sterile flat surface. A sterile, lint-free cotton bud dipped in 0.5 M NaOH solution was used to rub the stretched HAMs. It is possible to visualise cellular debris being removed from the HAM surface. The HAM was rubbed until there was no further visible debris being removed from the HAM surface. The HAM was then washed twice in PBS and once in LEC culture medium and immediately used for culture.

### 2.4 LSC ex vivo explant culture

Sixteen limbal explants were fashioned from a single corneoscleral ring that was cut into 16 equal segments. The corneoscleral ring was obtained from the Manchester Eye Bank with a service level agreement with the Newcastle upon Tyne Hospitals NHS Foundation Trust, UK after donor informed consent for research. A decision was made to use only 1 corneoscleral ring to eliminate bias from using different rings for different cultures, which could have affected LSC culture. Whilst acknowledging the fact that LSCs are more abundant at the 12 o'clock and 6 o'clock positions at the limbus, to try to eliminate bias from this, one corneoscleral ring was cut into 16 equal limbal segments, and the 16 pieces kept together in culture medium. The limbal explants were then plated in a random fashion on each of the 16 HAM constructs, without any preference for the location in which they came from, i.e. the operator and investigator was masked to where each explant came from. In this way, whichever limbal explant segment was plated on each HAM construct was completely random, minimising any bias from this. HAM constructs were prepared by stretching out and trimming the HAM, followed by wrapping and trapping its edges between two glass coverslips and placing in a 35mm culture well of a six well plate. Limbal explant cultures were established in quadruplicate (n = 4) on each type of HAM substrates: Group 1, fresh HAM (control); Group 2, HAM decellularised with 0.5 M NaOH; Group 3, HAM decellularised with 0.5% SDS; Group 4, γ-irradiated HAM decellularised with 0.5% SDS. The limbal epithelial medium (3:1 DMEM:Ham's F12 supplemented with 10% (w/v) fetal bovine serum, hydrocortisone, insulin, triiodothyronine, adenine, cholera toxin and epidermal growth factor) was exchanged every two to three days, and explant outgrowth was marked on the underside of the six-well plate at every medium exchange. Once the explant outgrowth reached >90% confluence, the culture was terminated and for each group, one confluent culture was analysed histologically, including through immunocytochemistry. For the remaining confluent cultures, the *ex vivo* expanded LSCs were separated from the HAM substrates by incubation with 4 mL of Trypsin 0.05% (w/v) solution (Gibco, USA) for ten minutes at 37 °C. The number of viable cells was determined in a dual-chamber haemocytometer under a light microscope. The cells were re-suspended in 1 mL of limbal epithelial growth medium to be used immediately (e.g. in colony forming efficiency assays), or frozen at −80 °C for later use.

### 2.5. Assessment of growth rate

Limbal explant outgrowth expansion was marked on the underside of the culture well every two to three days at every medium exchange (Fig. 8). At the termination of the culture, the sequential outgrowth markings were transferred to an acetate sheet, which was then placed over a graph paper to calculate the area covered by the limbal explant outgrowth by counting the number of small squares and multiplying by 4 to give the total surface area in mm^2^ covered by expanded cells on the HAM. This was then plotted as a graph of the total surface area of the outgrowth (in mm^2^) against time in culture.

**Fig. 1:**
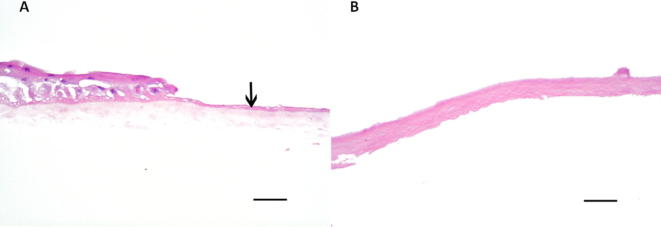
Histological micrographs with haematoxylin and eosin staining showing (A) fresh HAM, arrow denotes original amnion epithelium with limbal stem cell growth to the left of the image; (B) decellularised HAM. Scale bar is 500 µm.

**Fig. 2:**
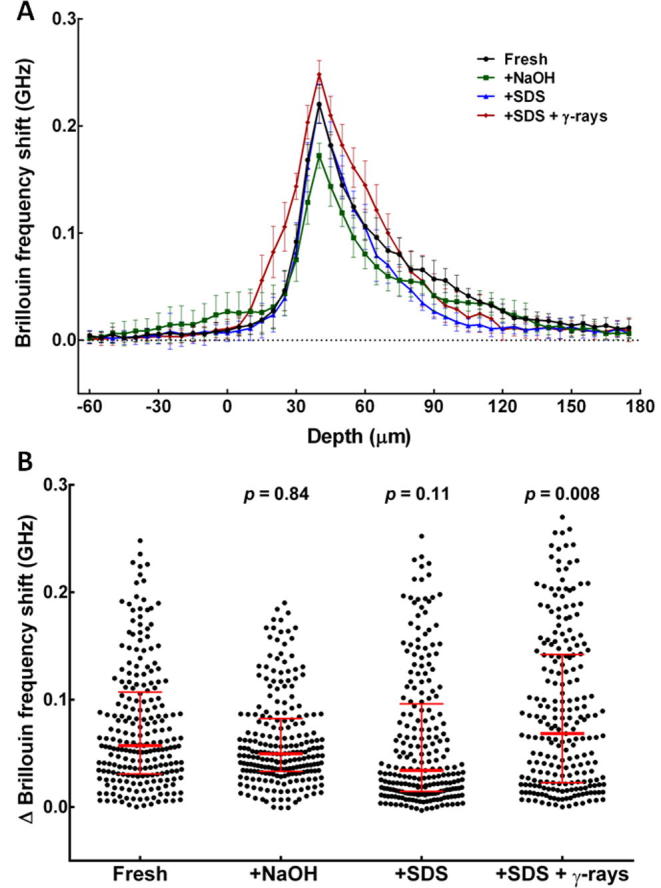
Brillouin shift measurements (GHz) in all four groups normalised against the surrounding liquid measurements; (A) graphical representation of stiffness measurements through all four groups, (B) scatter dot plot of normalised Brillouin frequency shift measurements from all four HAM tissue groups (median values and interquartile range are depicted in red; *p* values calculated by Kruskal-Wallis non-parametric test).

**Fig. 3:**
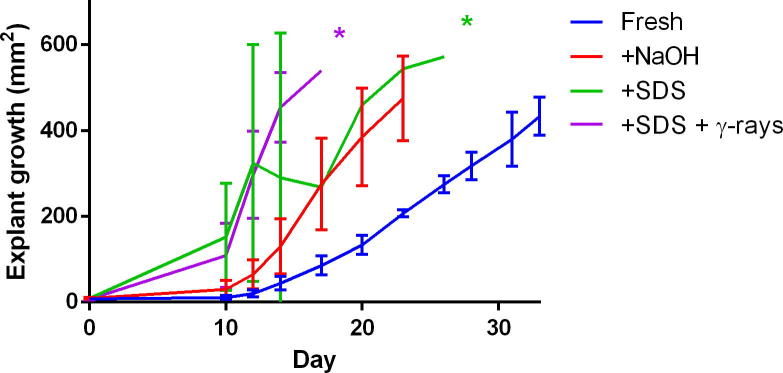
Graph showing mean outgrowth of limbal epithelial cells during explant culture in mm^2^ in all four groups (error bars are SD, ^*^p < 0.05), n = 4.

**Fig. 4:**
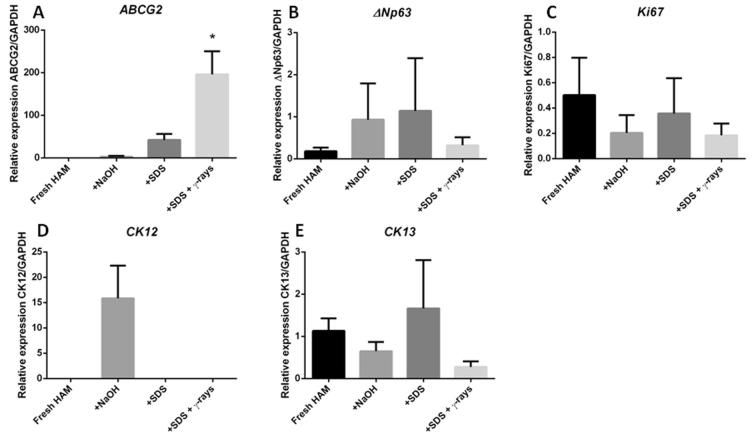
Graphs showing the RT-qPCR expression of (A) *ABCG2*, (B) *ΔNp63*, (C) *Ki67*, (D) *CK12* and (E) *CK13*, relative to *GAPDH* expression in all four groups (n = 3), * = *p* < 0.05.

**Fig. 5:**
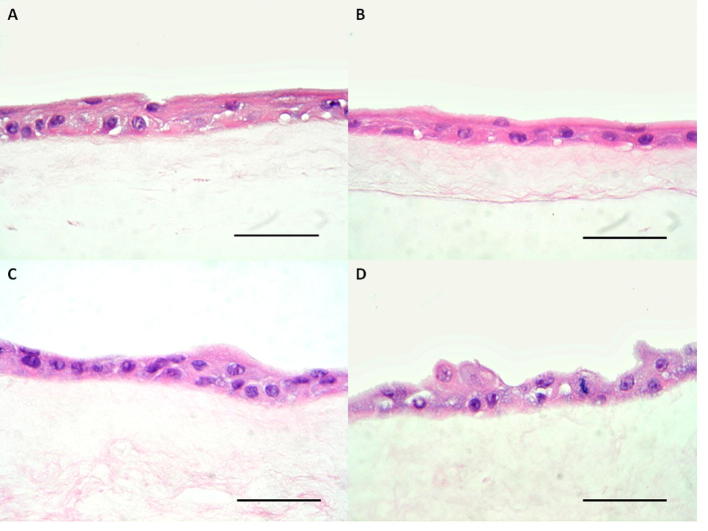
Haematoxylin and eosin stained histological images showing cell morphology of limbal explant cultures on (A) fresh HAM, (B) NaOH decellularised HAM, (C) non-gamma irradiated SDS decellularised HAM, and (D) gamma-irradiated SDS decellularised HAM. Scale bars are 100 µm.

**Fig. 6:**
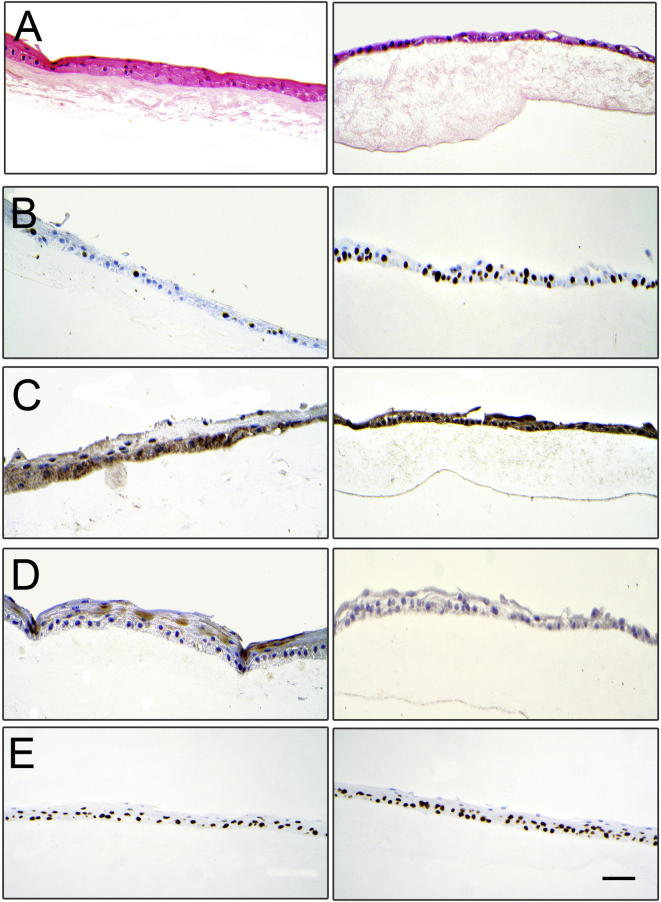
Histological images showing cultures on frozen non decellularised HAM in the left column and cultures on HAM decellularised with 0.5% SDS without γ-irradiation on the right column. (A) Haematoxylin and eosin stain, (B) Ki67 expression, (C) ABCG2 expression, (D) CK12 expression (E) ΔNp63 expression. Scale bar is 100 µm.

**Fig. 7:**
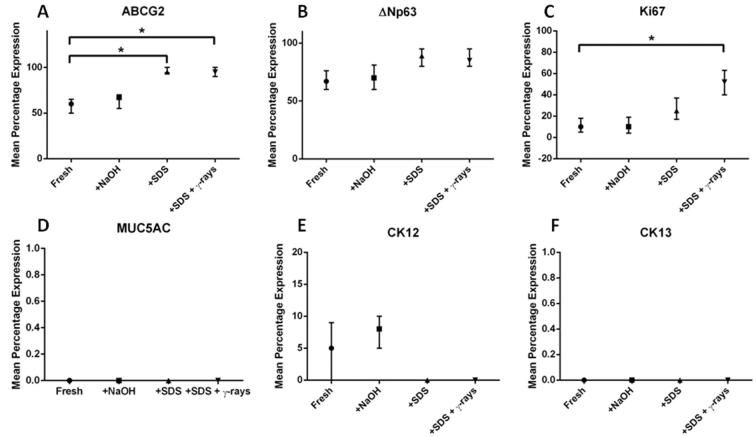
Graphs showing the median percentage expression of (A) ABCG2, (B) ΔNp63, (C) Ki67, (D) MUC5AC, (E) CK12, (F) CK13 in each group by immunocytochemistry (n = 3); error bars denote the interquartile range. The difference between the mean expression of each marker in each group relative to their expression on fresh HAM calculated by one-way ANOVA with Bonferroni *post hoc* analysis. * denotes *p* < 0.05.

**Fig. 8:**
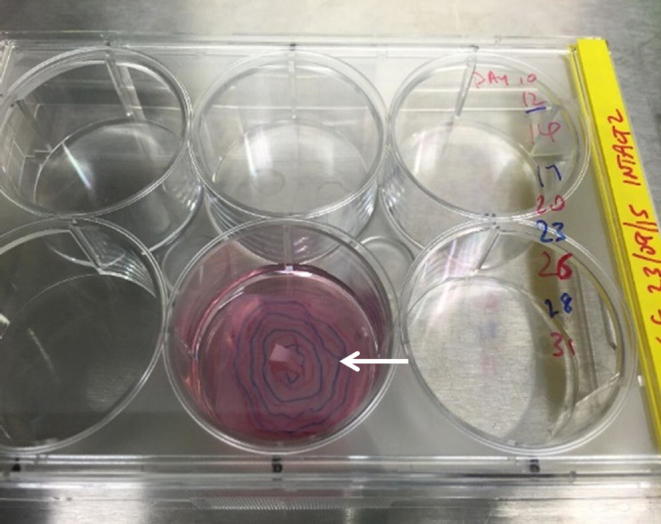
Colour photograph showing limbal explant in culture on human amniotic membrane construct. Outgrowth expansion was marked on the underside of the culture well (arrow).

### 2.6 Colony forming efficiency (CFE) assays

Colony forming efficiency (CFE) assays were used to determine the ability of limbal epithelial cells to form colonies. Briefly, cells from confluent limbal explant cultures were separated from HAMs by trypsinisation. Five hundred cells from each group were used in CFE assays, with three assays set up for each experiment group on a feeder layer of mitotically inactivated J2-3T3 mouse fibroblasts in six-well 35 mm cell culture plates. The CFE was measured on 12 days post-seeding. The culture was fixed in 3.7% (w/v) formaldehyde (VWR International, UK) in PBS and colonies were stained with 1% (w/v) Rhodamine B (Sigma-Aldrich, UK) in methanol for ten minutes at room temperature. Colonies were then counted under a dissecting microscope (SMZ645, Nikon, Japan) and CFE was calculated as the ratio between the number of colonies and the number of plated cells plated.

### 2.7. Histology and immunocytochemistry assessment

One limbal explant culture from each group was analysed histologically and by immunostaining. Histological analysis included an assessment of both the macroscopic and microscopic appearances of the HAM and the *ex vivo* expanded epithelial cells. The cells were stained by immunocytochemistry for putative limbal stem cell markers ΔNp63 (Dako Ready-to-Use pre-diluted [Agilent Technologies, Glostrup, Denmark]; antigen retrieval using Dako high pH 8 target retrieval solution) and ABCG2 (clone BXP-21 [Chemicon, EMD Millipore, Billerica MA, USA], used at a dilution of 1:50 with pH 6 retrieval using Bond ER1 [Leica Biosystems, Wetzlar, Germany]), a marker of corneal epithelium CK12 ([Abgent, San Diego CA, USA], diluted 1:100 in 0.2% calcium chloride buffer; Trypsin retrieval at pH 7.8), two markers of conjunctival epithelium CK13 ([Abcam, Cambridge, UK], dilution 1: 50, Trypsin antigen retrieval at pH 7.8) and MUC5 AC (clone CLH2 [Novocastra, Newcastle-upon-Tyne, UK] dilution of 1:100, with pH 6 retrieval using Bond ER1 [Leica Biosystems, Wetzlar, Germany]) and a marker of stem cell proliferation Ki67 (Dako Ready-to-Use pre-diluted [Agilent Technologies, Glostrup, Denmark]; antigen retrieval using Dako low pH 6 Dako target retrieval solution). Visualisation of staining was with avidin-biotin complex (ABC) (Vector Labs, Peterborough, UK) with diaminobenzidine brown reaction product. The percentage expression of a given marker was calculated from the immunostaining slides by counting 100 nuclei in the middle and at either end of the slides (3 fields in total), and the number of immune-positive cells were noted and an average percentage was calculated.

### 2.8 Quantitative reverse Transcriptase Polymerase chain reaction (qRT- PCR)

To confirm the quality of the expanded limbal epithelial cells, qRT-PCR of each culture group was performed. Quantitative RT-PCR is a reliable method of detecting and quantifying the expression of a particular transcript and represents the most sensitive method of determining differences in mRNA expression.

One hundred thousand (1 × 105) cells isolated from each experiment group were used in triplicate. Total RNA was extracted and cDNA was synthesised using the Cells-to-cDNA™ II kit (Ambion, Life Technologies, UK) as per the manufacturer’s protocol. Each reaction was set up using Go-Taq® qPCR Master Mix (Promega, USA) and was composed of 5 µL 2X Master Mix buffer, 0.4 µL forward primer, 0.4 µL reverse primer, 0.8 µL template cDNA, 3.7 µL RNAse-free water and 0.1 µL COX. All reactions were analysed on a Quantstudio™ 7 Real Time PCR System (Applied Biosystems™, USA) according to the manufacturer’s protocol using SYBR® Green as the detection dye, and ROX™ channel to detect COX as the reference dye for the entire plate. A standard, 40-cycle qPCR was performed for each sample, consisting of an activation cycle at 95 °C for two minutes, followed by 40 denaturation cycles at 95 °C for 15 s, an annealing/extension temperature of 60 °C for 60 s and dissociation cycle at 60–95 °C and two data acquisition steps. Each sample was analysed in triplicate, with *GAPDH* as the standard reference gene used and RNAse-free water as the negative control.

### 2.9 HAM stiffness

Brillouin spectromicroscopy was used to measure relative stiffness in the HAM in each group. This is a non-invasive, non-destructive method described by Lepert et al. that allows samples’ stiffness to be measured *in vitro*, in physiological conditions [31]. A single-stage virtually imaged phased array (VIPA) spectrometer was used to measure the Brillouin shift, which is directly related to the acoustic velocity. The samples were imaged one at a time, stretched on their nitrocellulose paper backing with a hole punched centrally so the reading could be made without interference from the nitrocellulose paper, immersed in PBS. For each sample, the Brillouin shift (GHz) was measured along the depth of the sample in steps of 5 µm, at nine locations spaced by 250 µm on a square grid. The Brillouin shift measurements taken across the material’s depth were represented in a scatter dot plot, with their corresponding median and interquartile range.

## 3 Results

A confluent limbal epithelial monolayer of cells was successfully established on 14 of the 16 explant cultures, on all four HAM substrate preparations (fresh HAM, HAM decellularised with NaOH, HAM decellularised with SDS and γ-irradiated HAM decellularised with SDS). A confluent monolayer was not established in one SDS decellularised HAM culture, and one fresh HAM culture. Fig. 1 shows haematoxylin and eosin stained sections of fresh HAM (A) and decellularised HAM (B). There was no subjective difference in HAM transparency after decellularisation and/or γ-irradiation. All HAM were freshly prepared by NHSBT and provided frozen in the same way.

### 3.1 HAM stiffness

The mechanical properties of HAM tissues were evaluated using Brillouin spectro-microscopy (Fig. 2). Brillouin frequency shift values obtained from each group of tissues were normalised against the average frequency shift from the surrounding medium (Fig. 2A, −60 to 0 µm region). Fresh HAM showed a maximum Brillouin frequency shift in the tissues’ anterior regions, followed by a sharp slope decrease in frequency shift in their posterior regions (Fig. 2A; black line). Decellularised HAM tissues showed a similar Brillouin profile (Fig. 2A). In addition, HAM decellularised with NaOH or SDS showed lower Brillouin shift values (Fig. 2A; green and blue line, respectively), however the ΔBrillouin frequency shift of fresh, +NaOH, and +SDS tissues (with median values of 0.057, 0.050, and 0.034 GHz, respectively) were not deemed significantly different (Fig. 2B). In contrast, tissues subjected to SDS and γ-rays treatment showed increased Brillouin frequency shift values (Fig. 2A; red line), which were significantly higher (p = 0.008) compared to those of fresh HAM (Fig. 2B; median of 0.068 GHz). Taken together, these results indicated that γ-irradiation marginally stiffened HAM tissues, whereas NaOH and SDS treatments had no substantial effect in HAM compliance.

### 3.2 Limbal explant outgrowth

Limbal explant cultures grew fastest on HAM decellularised with SDS followed by those cultured on HAM decellularised with NaOH and finally cultures on fresh HAM. Of the two groups of HAM decellularised with SDS, the γ-irradiated sub-group demonstrated the fastest rate of limbal explant outgrowth. The difference in the growth rate of both SDS groups was statistically significant compared with the fresh HAM group (Fig. 3, p < 0.05).

### 3.3 Cell phenotype

#### 3.3.1 qRT-PCR

There was no statistically significant difference between the substrate groups in terms of the expression of *ΔNp63, CK12, CK13 or Ki67* (Fig. 4). Limbal explants cultured on SDS decellularised HAM with γ-irradiation showed significantly higher expressions of *ABCG2* than those cultured on fresh HAM (p = 0.01, Fig. 4A). Although the groups showed a similar expression of *ΔNp63*, explants cultured on SDS decellularised HAM had the greatest expression, followed by those cultured on NaOH decellularised HAM (p > 0.05, Fig. 4B). All four groups showed a similar expression of *Ki67* (p > 0.05, Fig. 4C). Limbal explants cultured on HAM decellularised with NaOH showed a higher expression of *CK12* than limbal explants cultured on any other HAM preparation (p > 0.05, Fig. 4D), whereas in terms of *CK13* expression, all three groups showed a similar level of expression to those cultured on fresh HAM (p > 0.05, Fig. 4E).

#### 3.3.2 Immunostaining

There was no difference in cell morphology between LSCs cultured on the different substrates (Fig. 5A–D). There was no significant difference between the groups in terms of expression of ΔNp63, CK12, CK13 or MUC5AC, although only cells cultured on fresh HAM and NaOH treated HAM expressed CK12 (Fig. 6B–D). None of the cultures, in any group, expressed MUC5AC or CK13. The expression of ABCG2 (SDS, p = 0.0002 and SDS +γ-rays p = 0.0003) and Ki67 (SDS, p = 0.2214 and SDS +γ-rays, p = 0.0018) were significantly greater in SDS decellularised HAM compared with fresh HAM (Fig. 7A and C).

### 3.4 Colony forming efficiency (CFE) assays

Colony forming efficiency assays were performed in all 4 groups. After 12 days in culture, the CFE for the fresh HAM yielded a mean of 3.0% (85.1% holoclones), for the SDS-decellularised HAM this was a mean of 2.9% (83.3% holoclones) and for the SDS-decellularised HAM with γ-irradiation this was 2.8% (95.9% holoclones, p > 0.05). Colony forming efficiency assays carried out with cells expanded on NaOH decellularised HAM did not yield any colonies. The greater proportion of holoclones in the γ-irradiated HAM can be explained by the greater expression of limbal progenitor markers in these cultures. A plate showing the CFE assays with cells cultured on SDS decellularised HAM is shown in Supplementary Figure A.

## 4 Statistical methods

The difference in rates of limbal explant outgrowth was determined by two-way ANOVA. The difference between the groups in terms of the expression of *ΔNp63, ABCG2, Ki67, CK12* and *CK13* by qRT-PCR, as well as of the Brillouin frequency shift measurements, was analysed by Kruskal-Wallis non-parametric tests. The difference in expression of ΔNp63, ABCG2, Ki67, MUC5AC, CK12 and CK13 by immunocytochemistry was calculated by one-way ANOVA relative to the control group (fresh HAM), with Bonferroni *post hoc* analysis. Data was analysed using a statistical software package (Prism 6, Graphpad Software Inc, USA). Statistical significance was determined by *p* < 0.05.

## 5 Discussion

In the last couple of decades, there have been major leaps forward in tissue engineering to repair or replace tissue function or whole organs using cells, biomaterials alone or in combination [35]. Decellularisation processes were designed to produce a biological scaffold onto which stem cells could be seeded that reduced the risk of immune rejection and disease transmission. This is particularly pertinent in our phase II clinical trial of patients with unilateral total limbal stem cell deficiency (TLSCD) treated with *ex* vivo expanded autologous limbal stem cell transplant (ALSCT) where the HAM is the only allogeneic product used in the manufacture of LSCs. As part of a process to refine our culture method, where we previously refined our culture medium [36], we wanted to minimise the risk of a host response by using decellularised HAM and minimise the risk of disease transmission by using γ-irradiated HAM and sought to determine whether using a decellularised and/or γ-irradiated substrate would have a detrimental effect on our product.

To date there have been successful studies demonstrating the clinical application of decellularised tissues in humans, including the transplantation of decellularised cadaveric trachea [37], [38], nerve allografts [39], [40], and skin dermis [41] while there is ongoing research in developing a decellularised scaffold for the transplantation of organs [42]. Other groups have attempted to use synthetic bio-functional scaffolds such as fibrin gels [43], [44], [45], whilst other synthetic substrates have been used *in vitro* [13]. Additionally, Hogg et al. demonstrated in 2015 that γ-irradiation is an effective method of terminal sterilisation in the production of decellularised skin dermis for direct allogeneic transplantation [34]. To further refine our culture method and improve the quality of the starting material, we sought to determine if HAM sterilisation by γ-irradiation would have a detrimental effect on the culture of LSCs.

To the best of our knowledge, this is the first study comparing the *ex vivo* expansion of limbal explants on different forms of HAM preparation as a substrate and the use of γ-irradiation as a potential additional sterilisation step. This study has demonstrated a significantly more efficient rate of *ex vivo* expansion of limbal explants cultured on decellularised HAM compared to fresh HAM. Specifically, HAM tissues subjected to SDS decellularisation and γ-irradiation showed to provide the most suitable substrate for expansion, followed +SDS and +NaOH tissues. This difference could be due to the stiffening of HAM tissues after irradiation, illustrated by the significant increase in frequency shift values determined by Brillouin spectro-microscopy. The Brillouin frequency shift can give an acoustic measure of substrate stiffness and has been demonstrated by Lepert et al. to be an accurate method of determining stiffness in corneal tissues [31]. Using this method, we also demonstrated that the decellularisation process did not significantly affect the stiffness of HAM tissues, which might explain the more discrete influence of these treatments on the *ex vivo* expansion rate. Taking into account the rate of cell outgrowth from the limbal explants, the new, faster method of decellularisation (rubbing with NaOH solution) described by Saghizadeh et al. in 2013 did not appear to be as efficient as decellularisation using SDS. This was possibly due to the fact that the stiffness of NaOH-treated HAM tissue was similar or even slightly lower than that of fresh tissues. This notion is supported by Molladavoodi and colleagues’ work demonstrating that corneal epithelial cells had reduced rates of migration in compliant tissues [30].

Importantly, the Brillouin spectro-microscopy analysis showed that the frequency shifts of all HAM groups fall within the range reported in the limbus, which in turn has significantly lower shift values compared to the centre region of human and bovine corneas [31]. This and other studies [26], [29] have supported the hypothesis that the higher compliance (i.e., softness) of the limbus matrix plays a crucial role in LSC maintenance, whereas the stiffer central region of the cornea promotes epithelial cell differentiation. In this context, our data indicates that all different HAM groups have suitable mechanical properties to serve as medium- to long-term substrates for LSC culture. Conversely, it indicated that the stiffening effect of γ-irradiation was very slight, and far below the level that would compromise the use of HAM tissue for LSC culture.

The tensile strength of the HAM tissue could also be assessed, however, it has been demonstrated that Brillouin spectromicroscopy represents a method with great rigour and versatility and prone to less error. Previously, Lepert et al. have demonstrated Brillouin spectro-microscopy to be an effective method of evaluating the mechanical properties of collagen-based biological tissues and materials [31]. In particular, they were able to characterise the different regions of the cornea (centre, periphery, and limbus), using an all-optical, true non-contact technique. In the present study, this allowed us to determine the mechanical properties across the depth of different regions of HAM tissues while avoiding several possible technical artefacts (e.g. the influence of specimen shape and hydration, the geometry of the test setup, etc.). Ultimately, this allowed us to analyse multiple locations of the tissues, and thus provided us with data on both intra- and inter-tissue variations, as well as their possible impact on tissue performance.

This conclusion was supported by the analysis of the expression of stem cell and differentiation markers at transcriptional and protein levels. Through using immunocytochemistry to analyse our cultures, we were able to discriminate between non-specific and specific staining and perform an accurate assessment of these markers' expression in our cultures. For this reason, we chose immunocytochemistry over western blotting. We also performed qRT-PCR as an additional and more accurate method for validating gene expression. Our data confirmed that all decellularisation treatments did not compromise the potential of HAM tissues to serve as substrate for limbal epithelial stem cell maintenance and expansion. In particular, HAM subjected to SDS and γ-rays were shown to preserve the expression of putative stem cell and cell proliferation markers ABCG2, as well as a lower expression of cell differentiation marker CK12 compared to fresh HAM. It should be highlighted that ABCG2 results are similar in qPCR and ICC, showing highest expression in SDS and γ-irradiated SDS decellularised HAM. However, this was not the case for Ki67, where no significant difference could be seen at qRT-PCR. We know that Ki67 undergoes post-translational modifications [46], [47] therefore we rely mostly on ICC data which interprets protein expression. Our results suggest that SDS-decellularised HAM can provide several advantages for the culture of limbal explants, both in terms of growth rate and preservation of corneal epithelial cell phenotype (i.e., by preventing cell differentiation). In addition, this method can potentially mitigate the risk of inducing a host response or disease transmission due to the cellular component of the substrate; however, this potential requires further investigation. To this purpose, γ-irradiation will enable the HAM substrate to be sterilised without compromising the *ex vivo* expansion rate from limbal explants, nor the LSC phenotype. However, it must be borne in mind that, there are many factors potentially involved in the preservation of stem cell properties in LSC culture, notably growth factors, matrix or other basement membrane proteins, and a novel matrix component termed heavy chain (HC)-hyaluronan (HA)/pentraxin 3 (PTX3) as the key soluble factor to maintain quiescence of limbal progenitor cells [48], [49], [50]. It is important, therefore, to perform future studies to investigate how decellularisation affects growth factor release and ECM composition. Ideally, HAM with minimal manipulation would be used, so as to preserve ECM-growth factor interactions, however this would be impossible in clinical transplantation studies, not least because the donor has to be screened for transmissible diseases at the time of donation and the tissue has to be screened after a period in quarantine, and only used for transplantation when both samples are negative.

Colony forming efficiency assays carried out with cells expanded on NaOH decellularised HAM did not yield any colonies, despite carrying out assays 3 times using these cells. This would imply that NaOH treatment may affect HAM growth factor and ECM composition resulting in impaired ability to give rise to CFE and that this substrate may not be suitable for LSC culture. Label retention studies can be performed and are useful for demonstrating the presence of slow-cycling cells. However, these assays are best performed *in vivo* enabling assessment of stem cell division in their native location over longer time intervals. *Ex vivo* stem cell expansion occurs within short-term cultures and in the presence of growth factors which accelerate stem and progenitor cell proliferation which marks true label retention in our experience. In view of this, we chose to perform qPCR and ICC to assess the expression of putative LSC markers.

In 2009, Shortt et al. compared the growth of LSCs isolated and grown by single cell suspension on feeder layers of 3T3-J2 cells before being seeded onto one of four different groups of HAM: 1) Intact (fresh) HAM; 2) Partially decellularised (scratched) HAM; 3) Fully decellularised HAM (SDS 0.03%; w/v); and 4) Fully decellularised HAM (SDS 0.03%; w/v) and peracetic acid-sterilised HAM [22]. Their study is an interesting one that compared four similar groups of HAM preparation to ours, however they use a different method of cell culture (cell suspension versus limbal explant cultures in our study); in our previous experiments, cell morphology was better when LSCs were cultured on HAM substrates than in cell suspension (data not published). Shortt et al. also used a different concentration of SDS (0.03% versus 0.5%). It is obvious that higher concentrations of SDS have the potential to cause damage to the tissue structure or basement membrane proteins, and if there is residual SDS, this may affect the biocompatibility of the tissue. However, in NHSBT's experience, 0.5% was found to result in a great degree of consistency in terms of cell fragment removal and therefore a better decellularisation process. Our own data also suggests that the scaffold is not cytotoxic and is biocompatible. NHSBT also has evidence that scaffolds produced using 0.5% SDS concentration is not significantly different to those produced using 0.03% SDS (Rooney P., personal communication). Similarly to our experiments, the authors found that the decellularised preparations resulted in the most confluent growth at 21 days in culture. In contrast to our findings, however, they found no significant difference in the expression of ΔNp63 or ABCG2 between the HAM preparations. In this study we have demonstrated a significantly increased expression in ABCG2 in cells cultured on HAM decellularised with SDS. In fact, Shortt et al. concluded that although limbal epithelial cells cultured on decellularised HAM demonstrated a more rapid growth, their morphology indicated they were more differentiated (well-stratified layers of lower density cells) than those cultured on fresh HAM (confluent monolayer of higher density cells). Their findings are supported by previous studies by Grueterich et al. (limbal explant cultures) and Koizumi et al. (cell suspension cultures) who came to similar conclusions when investigating LSC growth on decellularised HAM [20], [21].

It is accepted that the quality of HAM may vary between donors, and this was a primary reason for doing these experiments. However, one limitation of this study is that NHSBT, who provided all HAM used in these experiments, could not provide us with information as to whether all HAM came from the same donor as the information is not routinely available.

## 6 Conclusions

Similarly to previous reports, we have demonstrated that *ex vivo* expansion of limbal epithelial cells using the explant culture system occurs at a faster rate on decellularised HAM compared with fresh HAM. Using Brillouin spectro-microscopy, we have shown that the frequency shifts of HAM tissues are, even after the process of decellularisation or sterilisation, similar to that of the human corneal limbus. As such, the mechanical properties of HAM tissues showed to be suitable for LSC maintenance. Moreover, LSC differentiation does not appear to be influenced primarily by HAM substrate stiffness; however HAM stiffness did appear to affect cell migration rate. This leads us to conclude that SDS decellularised HAM may be more efficacious as a substrate for the *ex vivo* expansion of limbal epithelial cells for use in clinical trials, and in particular, the use of a γ-irradiated decellularised HAM allows the user to start the manufacturing process with a sterile substrate, making it potentially safer.

## Figures and Tables

**Supplementary Fig. 1:**
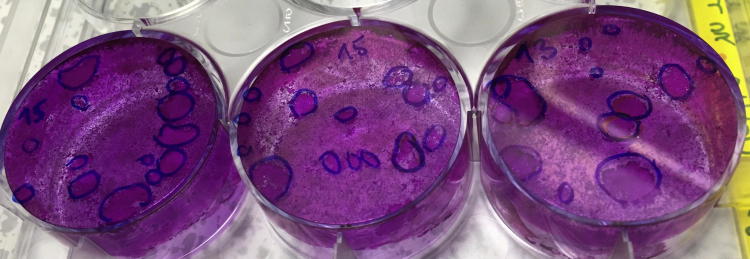
Colour photograph showing the colony forming efficiency (CFE) assay plates stained with Rhodamine B.
